# Research Progress on the Damping Mechanism of Magnesium Alloys

**DOI:** 10.3390/ma16237318

**Published:** 2023-11-24

**Authors:** Jinxing Wang, Zhicheng Wan, Cong Dang, Yi Zou, Jingfeng Wang, Fusheng Pan

**Affiliations:** 1 College of Materials Science and Engineering, Chongqing University, Chongqing 400030, China; 202209021059@stu.cqu.edu.cn (Z.W.); dangcong_24@163.com (C.D.); 202209021040@stu.cqu.edu.cn (Y.Z.); jfwang@cqu.edu.cn (J.W.); fspan@cqu.edu.cn (F.P.); 2 National Engineering Research Center for Magnesium Alloys, Chongqing University, Chongqing 400030, China

**Keywords:** magnesium alloys, damping mechanisms, influencing factors, damping capacity

## Abstract

Magnesium alloys with high damping, high specific strength and low density have attracted great attention in recent years. However, the application of magnesium alloys is limited by the balance between their mechanical and damping properties. The strength and plasticity of magnesium alloys with high damping performance often cannot meet the industrial requirements. Understanding the damping mechanism of magnesium alloys is significant for developing new materials with high damping and mechanical properties. In this paper, the damping mechanisms and internal factors of the damping properties of magnesium alloys are comprehensively reviewed. Some damping mechanisms have been studied by many scholars, and it has been found that they can be used to explain damping performance. Among existing damping mechanisms, the G-L dislocation theory, twin damping mechanism and interface damping mechanism are considered common. In addition, some specific long-period stacking ordered (LPSO) phases’ crystal structures are conducive to dislocation movement, which is good for improving damping performance. Usually, the damping properties of magnesium alloys are affected by some internal factors directly, such as dislocation density, solute atoms, grain texture and boundaries, etc. These internal factors affect damping performance by influencing the dissipation of energy within the crystal. Scholars are working to find novel damping mechanisms and suitable solute atoms that can improve damping performance. It is important to understand the main damping mechanisms and the internal factors for guiding the development of novel high-damping magnesium alloys.

## 1. Introduction

With the development of modern industry, the damage caused by the vibration and noise of components is becoming more and more serious. Vibration and noise have gradually become major public hazards [1]. According to failure analysis, most of the equipment damage in the military industry and aerospace fields is related to vibration. In life, the noise of household appliances and the damping performance of automobiles are closely related to people’s safety [2,3]. After research on noise propagation and vibration generation, scholars have found that vibration reduction using materials is effective. Developing a kind of high-damping metal material with excellent comprehensive properties has always been a goal pursued by scholars.

Damping refers to mechanical energy dissipation caused by the material itself [4]. Because of their good application prospects, metal damping materials can be used as structural and functional materials. Magnesium (and its alloy) is the lightest structural material (its density is 1.7 g/cm^3^) and one of the metal materials with the best damping performance [5]. In addition, magnesium also possesses the advantages of rich reserves, electromagnetic shielding, high specific strength, etc. These advantages make damping magnesium alloys widely used in aerospace, transportation and electronic equipment [6,7,8]. Good mechanical properties are important for workpieces operating under cyclic loads. However, due to the contradiction between damping properties and mechanical properties, it is usually difficult for the damping properties and mechanical properties of existing magnesium alloys to meet the requirements of use at the same time [9]. On this basis, scholars have conducted a lot of research to achieve a balance between the damping and mechanical properties of magnesium alloys. The research on the damping properties of magnesium alloys is scattered, and the damping mechanisms of magnesium alloys have not been explained completely. Therefore, it is necessary to systematically discuss the mechanisms and internal influencing factors of the damping performance of magnesium alloys. Combining various damping mechanisms has become a basic principle in designing magnesium alloys with high damping and high mechanical properties. Determining how to design the crystal structure and defects such that a material is beneficial to damping performance is the focus of recent research [10,11].

## 2. Damping Mechanisms of Magnesium Alloys

The reason for the internal friction of a material is that the strain always lags behind the stress during deformation [12]. Regarding the sources of internal friction, the damping mechanisms of magnesium alloys are generally divided into dislocation damping, grain boundary damping and twin damping. In reality, the damping mechanisms in metals are complex and diverse. So, they usually influence the damping properties of metals via one or more mechanisms. The dislocation damping mechanism at low temperatures generally dominate magnesium alloys. At high temperatures, thermal activation will lead to grain boundary sliding. Then, grain boundary damping or twin damping becomes the main damping mechanism of magnesium alloys [13,14]. 

### 2.1. Dislocation Damping Mechanism in Magnesium Alloys

**Figure 1:**
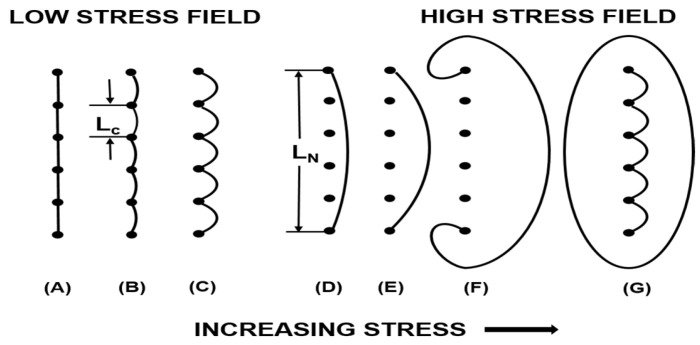
Schematic diagram of G-L dislocation theoretical mode: (**A**–**C**) the process of dislocation movement between two weak pinning points as the stress increases; (**D**,**E**) the process of unpinning movement of a dislocation; (**F**,**G**) The process of dislocation movement between two strong pinning points as the stress increases [16,17].

Dislocation is a phenomenon whereby one or several rows of atoms in metal are staggered. Many dislocations exist in the crystal as energy consumption, and this is the main reason for the high damping performance of magnesium alloys. The density, length, and distribution of dislocations significantly influence the damping properties of magnesium alloys. As early as 1940, Read [15] proposed that dislocations in crystals would dissipate energy. After that, Granato and Lücke [16,17,18] proposed a dislocation pinning model, as shown in Figure 1.

Dislocation damping comes from dislocation motion under external force. The G-L dislocation theory holds that both strong and weak pinning points pin dislocations. When the strain amplitude is low, dislocation damping is resonant internal friction independent of amplitude [19]. Currently, the vacancy and solute atoms, etc., generally act as weak pinning points in the crystal, and then, the weak pinning points will pin the dislocation. Under external stress, there is a reciprocating bow-out motion between weak pinning points. This process will dissipate energy. When the strain amplitude is large, dislocation damping is a static hysteria-type internal friction related to the strain amplitude. Grain boundaries, the second phase, and dislocation entanglement are regarded as strong pinning points. When the critical strain amplitude is beyond, the dislocation will have an “avalanche” movement of unpinning at the weak pinning points and continue to be pinned by strong pinning points.

Generally speaking, the damping properties of magnesium alloys are divided into strain-amplitude-independent damping (Q_l_) and strain-amplitude-dependent damping (Q_h_). Under low stress, the length between dislocation segments is expressed by L_c_, and the following formula can express the internal friction caused by overcoming resistance during movement:

(1) $$Q_{l}^{-1}=\rho A\omega L_{c}^{4}/(36Gb^{2}).$$

In the Formula (1), ρ is the density of movable dislocation, L_c_ is the length between weak pinning points, G is the shear modulus, ω is the angular frequency, b is the Burger vector, and A is a constant. It can be seen that in the low-strain region, the larger the length L_c_ between dislocation density ρ and weak pinning points, the better the damping performance of the alloy. And the number of weak pinning points should be reduced appropriately, and a higher dislocation density should be introduced. At high strain amplitudes, the unpinning motion of dislocation consumes a large amount of energy and produces large damping. The distance between dislocations changes from L_c_ (distance between weak pinning points) to L_n_ (distance between strong pinning points). At this time, the internal friction equation of strain amplitude can be written as:

(2) $$Q_{h}^{-1}=(C_{1}/\varepsilon )\exp(C_{2}/\varepsilon ).$$

In the Formula (2), $C_{1}=\left(\frac{\rho L_{n}^{3}}{L_{c}^{2}}\right),\;C_{2}=(1/L_{c})$, ε is the strain amplitude, and L_n_ is the distance between strong pinning points. It can be seen that the damping performance of the alloy is inversely proportional to the length L_n_ between strong pinning points, and increasing the number of strong pinning points can effectively improve the damping performance of the alloy.

**Figure 2:**
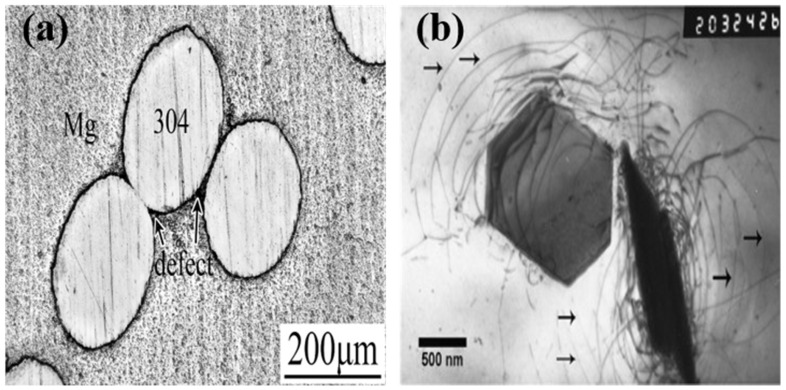
Some novel microstructures in magnesium alloys: (**a**) microstructure of the interface of 304/Mg [25]; (**b**) TEM image of as-cast alloy (the long and parallel dislocations are indicated by arrows) [26].

Uhríčik et al. [20] found that the temperature damping of magnesium alloys can be divided into temperature-independent and temperature-dependent regions. Among them, the temperature-dependent region is characterized by a maximum damping value and the exponential growth of damping performance at higher temperatures. The reason for this phenomenon is continuous precipitation. The finer and more numerous the precipitates, the more obstacles there are to dislocation movement, and the damping value reaches a maximum value. Zhang et al. [21] studied the influence of different heat treatment processes on the damping properties of AZ91D alloys and found that the G-L dislocation theory can explain these phenomena well. Solution strengthening makes the second phase in the alloy dissolve. According to the G-L dislocation theory, the movement of dislocations is less restricted, and the damping performance of the alloy is improved. The aging process promotes the precipitation of the Mg_17_Al_12_ phase, making dislocation more difficult to move. So, the alloy’s damping performance is reduced. Mart’ınez. et al. [22] found that the damping properties of AZ31, AZ61 and AZ91 alloys decreased with increased Al content. The addition of the Al element undoubtedly firmly pins dislocations, which reduces damping performance. Wan et al. [23] found that G-L dislocation theory can significantly explain the strain-amplitude-dependent damping spectrum under a low or moderate strain amplitude. Microplastic deformation occurs under high strain amplitude, but the deformation is still predominated by the movement of dislocations. Parande et al. [24] added eggshell particles to a Mg-Zn matrix and found a mismatch of them, leading to the multiplication of dislocations. Damping properties were improved because of the higher density of dislocation. Li et al. [25] found a novel microstructure, shown in Figure 2a, by mixing 304 stainless steel wire with Mg (304/Mg). The high density of dislocations around the 304 stainless steel wire could be an energy dissipation source, which led to a higher damping capacity of 304/Mg.

Wang et al. [26] developed an as-cast Mg-3Cu-1Mn-2Zn-1Y alloy and found an abnormal damping capacity increase when the Y and Zn content rose. They attributed this to the long and parallel dislocations and second phase with a novel morphology. The second phase formed because of the extreme discrepancy between the thermal expansivity of the second phase and Mg matrix, as shown in Figure 2b. Regarding the G-L dislocation theory, the long and parallel dislocations increased the volume of ρ (dislocation density), L_N_ (the length of strong pinning points) and L_C_ (the length of weak pinning points); all of these factors will lead to a higher damping property.

**Figure 3:**
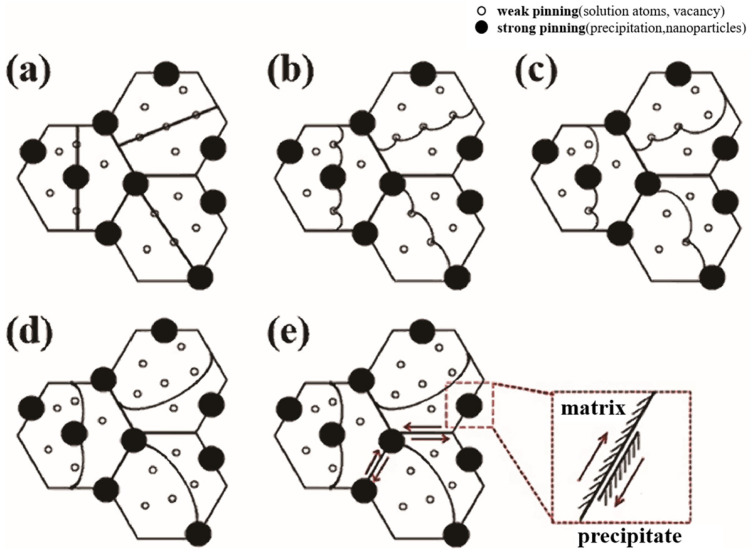
Schematic illustration of the temperature-dependent damping mechanism for the CEC-processed 1%SiCnp/AZ91D composite: (**a**) unloaded, (**b**) low-temperature zone, (**c**) intermediate-temperature zone I, (**d**) intermediate-temperature zone II and (**e**) high-temperature zone [29].

Yu et al. [27] added Ti_2_AlC phase to a magnesium matrix, and the magnesium alloy’s damping performance increased by 20% by volume. However, above 200 °C, the damping performance of the magnesium alloy increased significantly due to interfacial sliding. This enhancement of damping performance can be explained by the fracture mode of the alloy changing from the delamination of Ti_2_AlC to interfacial debonding at 200 °C. Wu’s group [28] also discovered that the dislocation damping mechanism dominated from room temperature to 250 °C in an as-cast SiCp/Grp/AZ91 composite. The high density of dislocations due to the different coefficients of thermal expansion is the key to a better damping capacity. Ebrahimi et al. [29] studied the damping mechanism of a CEC (cyclic extrusion and compression)-processed 1%SiCnp/AZ91D composite with increasing temperature. An illustration of the damping mechanism is shown in Figure 3. They also verified energy dissipation mainly caused by the vibration of dislocations between pinning points.

Ma et al. [30] found that the G-L theory can explain the damping mechanism well under the critical temperature by controlling the extrusion parameters. They demonstrated that the mechanical properties and damping properties of alloys with low temperature and low extrusion rates can reach the application standard. Tian et al. [31] indicated that under cyclic shear stress, the damping performance curve of AZ61 first increases, and then, decreases. In the first and second stages of low pressure, the rise in damping performance is linear and it can be explained by the G-L theory. Then, the higher shear stress leads to dislocation multiplication and increased movable dislocation density. After that, dislocation pile-up will lead to a decrease in damping performance. 

Since the G-L dislocation theory emerged in the 1950s, many scholars have conducted in-depth research on it. While the G-L dislocation theory has been widely accepted, researchers have found that it has shortcomings. For example, when the strain amplitude is large, all the dislocation lines come off the nail, and the strain energy always rises, but the area enclosed by the hysteric loop of the stress–strain curve remains unchanged. Therefore, theoretically, the damping value of the magnesium alloy should decrease and reach a maximum value. However, practical experimental results show the damping value of the alloy keeps rising, which is inconsistent with the G-L theory. In addition, in a large stress–strain environment, the damping test data are obviously inconsistent with the G-L dislocation theory. The above defects of the G-L dislocation theory have prompted scholars to study magnesium alloys’ damping mechanisms further.

**Figure 4:**
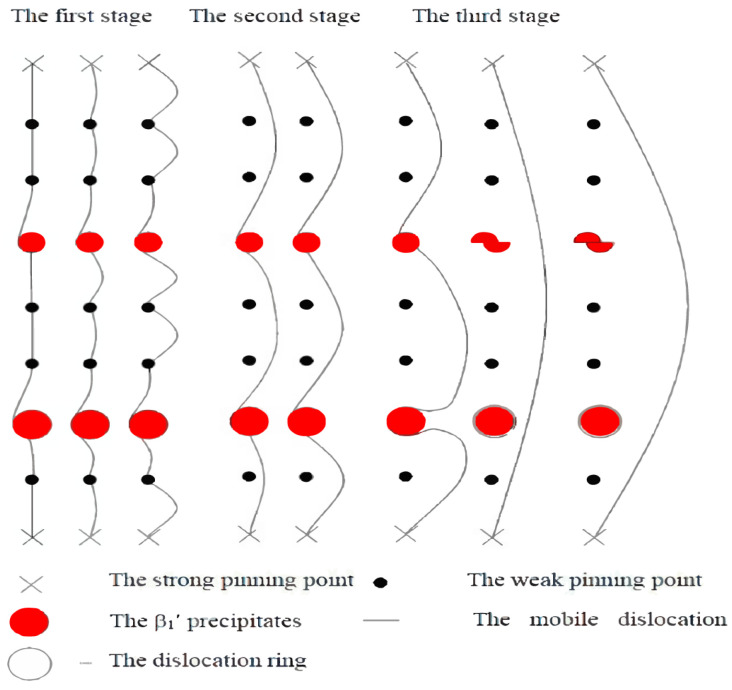
The modified Granato–Lücke model [33].

Zhou et al. [32] proposed an improved G-L model. After aging treatment, a ZK60 alloy showed higher strain-amplitude-dependent damping performance. This phenomenon was related to dislocation movement in the G.P.I zone. In the high-stress stage, dislocations bow out between strong pinning points and dissipate more energy by sweeping the G.P.I zone region. Similar results were also found in the research of Zhou’s group [33]. They also found that the β_1_′ phase (MgZn_2_) in ZK60 can improve magnesium alloys’ mechanical and damping properties. The mechanism is shown in Figure 4. Dislocation sweeps the β_1_′ phase to dissipate extra energy, showing an increase in the magnesium alloy’s damping performance.

Anasori et al. [34] found that the high damping of Ti_2_AlC itself is the main reason for the high damping performance of Ti_2_ALC-Mg composites upon adding TiC and Ti_2_AlC particles to a magnesium alloy, and. J. Göken et al. [35] found that cracks may also be used as damping sources to increase the damping properties of magnesium alloys. The diffusion process at the crack tip is considered the main mechanism of crack-induced damping. The damping performance of the alloys improves when the crack opens and decreases when the crack expands. Wu et al. [36] found a P_3_ damping peak in extruded Mg-11.2Li-0.95Al-0.43Zn at 215 °C and low frequency. The XRD experiments showed that the appearance of the P_3_ peak is related to recrystallization process and grain growth, but the specific mechanism needs further study.

Usually, in the process of forging magnesium alloys, a strong basal plane structure parallel to the machining direction will be obtained, further limiting the formability and anisotropy of the mechanical properties of magnesium alloys [37,38]. However, Wang et al. [39] found that the texture of an AZ31 magnesium alloy was suppressed during WAAM-GTAW (wire and arc additive manufacturing–gas tungsten arc welding) processing. A higher dislocation density was introduced to the grain when the texture changed. The above methods inspired us to develop magnesium alloys with high damping and mechanical properties. 

Since the 1950s, scholars have conducted much research on the G-L dislocation theory. The G-L dislocation theory can effectively explain the damping properties of magnesium alloys at room temperature and low strain. In recent years, the G-L theory has also become the mainstream damping mechanism of magnesium alloys. However, with the deepening of research, more and more phenomena contrary to the G-L theory have been discovered. This has led researchers to begin to explore a more comprehensive damping mechanism of magnesium alloys.

### 2.2. Boundary Damping Mechanism in Magnesium Alloys

The strength of magnesium alloys decreases sharply at high temperatures, which is usually attributed to grain boundary sliding [40,41,42]. However, in recent years, many scholars have found that the grain boundaries of magnesium alloys will dissipate extra energy when sliding. So, grain boundaries may be an energy dissipation source in magnesium alloys [43,44]. It is important to study the effect of grain boundary sliding on the damping properties of magnesium alloys. This inspired us to develop magnesium alloys with balanced damping properties and mechanical properties.

Cui et al. [45] proved the influence of twin boundary mobility on the damping capacity of magnesium alloys. They systematically studied the effect of annealing on the damping capacity of a pre-deformed magnesium alloy. After a short duration of annealing, the damping performance of the AZ31 magnesium alloy was improved. Still, after a long duration of annealing (5000s), the damping performance of AZ91 decreased because segregation and precipitation increased the stability of the twin boundary. As we all know, dislocations move faster than the speed of sound, and the rate of grain boundary movement is similar to the speed of sound [46]. The damping peaks appearing at different frequencies can directly reflect the respective contributions of dislocations and grain boundaries to damping. It is a feasible way to manufacture high-damping magnesium alloys by properly annealing. This can improve grain boundary mobility and thus improve the damping capacity of magnesium alloys.

Ma et al. [47] studied the influence of different heat treatments on the damping properties of a Mg-Zn-Y-Zr alloy (ZKW3 alloy). In a ZKW3 alloy as-cast and after annealing and T4 heat treatment, the dislocation damping mechanism was dominant. But after T6 heat treatment, the damping performance of the magnesium alloy was relatively the best because of the appearance of twins. The authors speculate that the movement of twin grain boundaries is the key to causing different damping performances. 

Somekawa et al. [48] studied the influence of deformation twins on the damping properties of magnesium alloys. They thought that the reason for the improved damping properties of this induced twin alloy might be the existence of many twin grain boundaries in the matrix and the increase in the average Schmidt factor. The strength of the magnesium alloy decreased sharply at high temperature, which is usually attributed to grain boundary sliding. Cui et al. [49] introduced twins into a magnesium alloy via short annealing at 250 °C. They found that the reciprocating motion of twins is the key to energy dissipation. After annealing, the alloy’s dislocation density decreases, further strengthening the effect of this reciprocating motion. This discovery may inspire the manufacture of new high-damping magnesium alloys.

To a certain extent, the grain boundary damping mechanism of magnesium alloys makes up for the shortcomings of the G-L dislocation theory. On the one hand, the relaxation of grain boundaries consumes energy at high temperatures; on the other hand, more grain boundaries means more fixed points. The fewer the grain boundaries, the larger the grain size, which is not conducive to the improvement of strength. Therefore, it is inefficient to control the damping properties of magnesium alloys using grain boundaries. This has forced scholars to conduct new research on shock-absorbing magnesium alloys.

### 2.3. Novel Discovery of Damping Mechanisms in Magnesium Alloys

Mg-RE (rare earth) alloys have become more and more popular because of their good mechanical properties and damping capacity. Some researchers have demonstrated that there is an LPSO phase by introducing rare earth elements to magnesium alloys [50]. They have also verified that LPSO is good for magnesium alloys’ mechanical properties and damping capacity [51,52,53,54].

**Figure 5:**
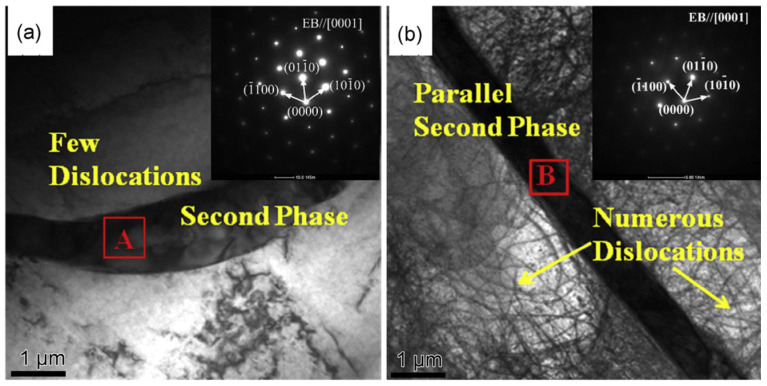
Bright-field TEM images of (**a**) Mg-1Ce and (**b**) Mg-2Ce alloys [56].

Lu et al. [55] found that different processes in Mg-Zn-Y-based alloys could obtain long-period stacked ordered phases. After heat treatment, obtaining a layered second phase in samples with fewer solid solution atoms in the matrix was more difficult. Moreover, the layered second phase significantly affects the damping properties of magnesium alloys. A sparse second phase can improve the damping properties without substantially reducing the mechanical properties of magnesium alloys. The reason may be that too-compact LPSO phases can hinder the dislocation motion in the matrix. The effects of different Ce contents on the microstructure and damping properties of a Mg-Ce binary alloy were studied by Wu et al. [56]. They found that Mg-2Ce’s damping performance is excellent in strain-amplitude- and temperature-dependent regions. A large number of parallel second phases were found in it, as shown in Figure 5. There are many parallel and uniform dislocations around this second phase, significantly improving the damping performance of magnesium alloys. 

Wang et al. [57] studied the effect of the LPSO phase in the specific alloy Mg-Cu-Mn-Zn-Y. The LPSO phase’s existence will hinder the dislocations’ motion and generation. Regarding the G-L theory, the damping property should have deteriorated, but it did not because of the presence of the LPSO phase. So, the authors speculated that there is some other mechanism of damping performance in magnesium alloys. Dang et al. [58] studied how the growth direction of the lamellar LPSO phase influences the damping performance of Mg-10Gd-2Y-1Zn-0.5Zr-0.2Nd alloys. They found that the growth direction of lamellar LPSO is the same as the movement direction of basal dislocation, which is the key to obtaining better damping properties.

**Figure 6:**
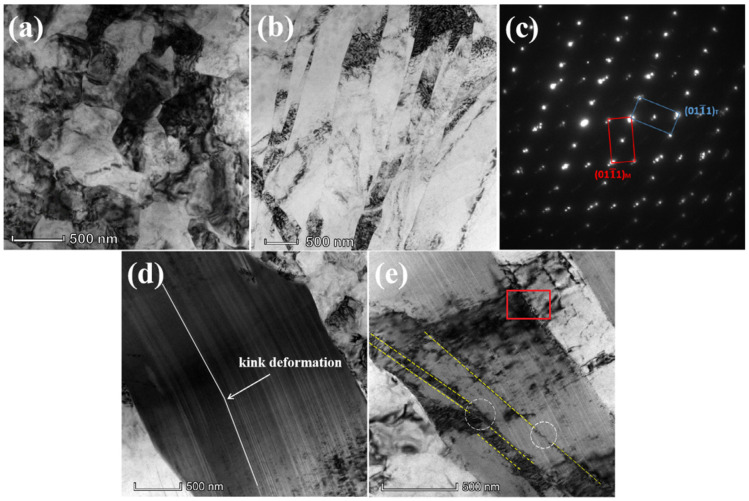
TEM images of the cold-rolled alloy after heat treatment: (**a**) bright-field image of the nano-grains; (**b**) bright-field image of the 1011 deformation twin; (**c**) SAED pattern from the twin boundary region in (**b**); (**d**) bright-field image of the 18R LPSO phase with kink; (**e**) bright-field image of the deformation twin in the 18R LPSO phase (the twin boundaries and a large number of dislocation regions are marked with dotted circles and red rectangles, respectively) [62].

Wang et al. [59] introduced micron and submicron SiCp into a magnesium matrix. They found that S-1+M-9 composite (0.2 μm 1 vol%+10 μm 9 vol% SiCp/AZ91) and M-10 composite (10 mm 10 vol% SiCp/AZ91) showed differences when controlling the average grain size to be consistent with the volume fraction of SiCp. This indicates that the part with a smaller grain size controls the damping performance of the magnesium alloy, that is, 1 vol% submicron SiCp. This may inspire us to study the damping mechanism of magnesium alloys further. Wu et al. [60] found that the high damping properties of graphite particles also improved the damping properties of magnesium matrix composites by adding graphite particles with different volume fractions into AZ91. However, when the volume fraction of graphite particles was higher than 15%, the damping performance decreased because Ti_2_AlC contains high dislocation density. A bimodal grain structure alloy consisting of fine recrystallized grains and coarse unrecrystallized grains was obtained by Wang et al. [61], who added manganese to a magnesium matrix in combination with low-temperature (165 °C, 180 °C, 220 °C and 300 °C) extrusion. On the one hand, the fine recrystallized grains are conducive to the activation of grain boundary slip and had good plasticity; on the other hand, due to a large number of movable dislocations in the unrecrystallized grains, it was conducive to the improvement of the damping capacity at room temperature and high temperature. Mg-8Li-4Y-2ER-2Zn-0.6Zr was heat-treated and cold-rolled, and a magnesium alloy with both damping and mechanical properties was obtained by Wang et al. [62]. After heat treatment, an LPSO phase was introduced into the alloy, and the damping performance of the alloy increased after cold rolling. As shown in Figure 6, the microstructure showed that the LPSO was twined and entangled. Generally speaking, the tangled boundary will hinder and pin the movement of dislocations, decreasing damping performance. However, this is different from the experimental results. On the one hand, the generation of the 18R LPSO phase reduced the base slip shear stress of the magnesium alloy; on the other hand, the reciprocating motion of the twin boundary dissipated energy. The interaction of twins and the LPSO phase leads to high damping performance of magnesium alloys.

Due to the new damping mechanism and suitable manufacturing process, some magnesium alloys containing SFE (stacking fault energy) show good damping and mechanical properties.

Yu et al. [63] studied the damping properties of four Mg-1at%X binary alloys (X = Zn, Sn, Ga, Al) and compared them with pure magnesium. He found that lattice distortion is the main reason affecting magnesium alloys’ strain-amplitude-independent damping performance. The SFE plays a leading role in strain-amplitude-dependent damping performance. The higher the SFE, the better the strain-amplitude-dependent damping performance. Xu et al. [52] made an as-extruded Mg-4Er-4Gd-1Zn alloy and an as-extruded Mg-8Er alloy through heat treatments. The Mg-4Er-4Gd-1Zn alloy is regarded as an excellent structural material because of the stacking fault of the basal plane with nanometer spacing and good mechanical properties. The Mg-4Er-4Gd-1Zn alloy possesses a smaller Schmidt factor and finer grain, but its damping performance is better than that of the Mg-8Er alloy. This further indicates that a stacking fault mechanism may affect the damping performance in magnesium alloys.

The study of damping mechanisms is an eternal topic in the field of the damping performance of magnesium alloys. Understanding the damping mechanism can not only help us control the damping properties of magnesium alloys, but also guide us to develop new high-damping magnesium alloys. The existing G-L dislocation theory, grain boundary damping mechanisms, and later, new damping mechanisms cannot fully explain the damping mechanism of magnesium alloys. In the future, the research on the damping mechanism of magnesium alloys will still be the focus of research.

## 3. Internal Influencing Factors of Damping Capacity of Magnesium Alloys

The dislocation density is the total length of dislocation lines in a unit-volume crystal [64]. The greater the dislocation density in the material, the greater the chance of dislocation starting. In addition, dislocation starting is also affected by grain orientation, and dislocation is easier to activate in grains with a more favorable orientation [65]. Regarding the G-L dislocation theory, dislocation is also affected by weak and strong nail points after it starting. Therefore, for magnesium alloys, the factors that affect the damping performance of magnesium alloys can be divided into external and internal factors. The external factors include strain amplitude, temperature, frequency and heat treatment process. The internal factors include dislocation density, solute atom grain orientation and grain boundary. The internal factors are mainly discussed here.

### 3.1. Dislocation Density

According to the G-L dislocation theory, the damping performance magnesium alloys is influenced by the crystal’s dislocation density directly. The higher the dislocation density, the more chance of the dislocation moving. The movement of dislocations between the pinning points dissipates energy, thus improving the damping performance of magnesium alloys. But a too-high dislocation density can easily produce dislocation entanglement, which also impacts the damping performance of magnesium alloys.

Liao et al. [66] found that dislocation density had a very significant effect on the damping properties of a magnesium alloy at either room temperature or high temperature. At room temperature, the addition of Si led to higher dislocation density of the Mg-9Al-xSi alloy. Some pinning points were scattered on the dislocation line, and the dislocations could move well. At high temperature, the better damping performance with increasing Si content is attributed to the higher dislocation density and grain refinement. Liu et al. [67] found that the solid solution state of a ZK60-2.8Nd Mg alloy possessed higher strain-independent damping compared to the as-cast and extruded states. On the one hand, the dynamic recrystallization during hot extrusion decreased the density of movable dislocations within the grains; on the other hand, the solid solution treatment increased the grain size slightly, and the dislocations were farther apart between the strong pinning points, improving the damping properties. In addition, the cast AZ31 Mg alloy was annealed and heat-treated at 673 K and 723 K. Kim et al. [68] observed the microstructure and grain texture, and found that the dislocation density of their sample was smaller and the damping performance was lower at 673 K. This indicates that the dislocation density of the Mg alloy directly affected the damping performance. Compared with the as-cast Mg-4Li-3Al-0.3Mn alloy, the damping performance of the extruded Mg-4Li-3Al-0.3Mn alloy was higher. Zhou et al. [69] found that the grains of the alloy became equiaxed after extrusion, and there were many dislocations in it. This was considered one of the reasons for the higher damping performance of the extruded Mg-4Li-3Al-0.3Mn alloy. Sometimes excessive dislocation entanglement also decreases the damping performance of Mg alloys. This phenomenon was observed and verified by Wang et al. [70]. Too much Mn content in Mg-Mn alloys means that the spacing between solute atoms is smaller and dislocations are entangled between the nodes. This is not conducive to dislocation movement, so the damping performance of the Mg-1.98%Mn alloy is significantly lower than Mg-1.71%Mn.

### 3.2. Solute Atoms

There are excellent damping properties in pure magnesium, but its mechanical properties limit the application of pure magnesium. In recent years, adding alloying elements to pure magnesium via solution strengthening has become an important method to improve magnesium alloys’ mechanical and damping properties [71]. On the one hand, solute atoms pin dislocations, making it more difficult for the dislocations to move; on the other hand, it will improve the mechanical properties of the alloy via a strengthening effect [72]. Therefore, it is very important to systematically understand the influence of solute atoms on the damping and mechanical properties of magnesium alloys to develop new magnesium alloys.

Jun et al. [73] studied the effect of Ca on the damping properties of an AZ91 magnesium alloy. In the strain-amplitude-independent region, the addition of Ca had little effect on the damping properties of magnesium alloys. The damping properties of the alloy treated with solution were better than those of as-cast alloys. In the strain-amplitude-dependent region, adding the Ca element could obviously improve the damping performance of the as-cast magnesium alloy. But the phenomenon observed in the magnesium alloy after solution treatment was the opposite. The aggregation of solute atoms may have caused this. Wan et al. [74] cast Mg-Ca binary alloys at 750 °C and 550 °C, respectively. The G-L dislocation theory can effectively explain the damping properties of both alloys. A nearly spherical α-Mg matrix can be observed in a Mg-1%Ca alloy cast at lower temperature, and its damping performance is also more excellent.

Ma et al. [75] compared the damping properties of an extruded Mg-Gd binary magnesium alloy at different temperatures, and found that Mg-1.5Gd showed good damping properties and mechanical properties at 360 °C. The damping properties can be explained by G-L dislocation theory. At low strain amplitude, the damping performance is affected by the length L_c_ between weak pinning points, SFE and dislocation density. Under high strain amplitude, the grain boundary acts as a strong pinning point to pin dislocations, so the damping performance of an as-cast Mg-Gd alloy with small grains is poor.

**Figure 7:**
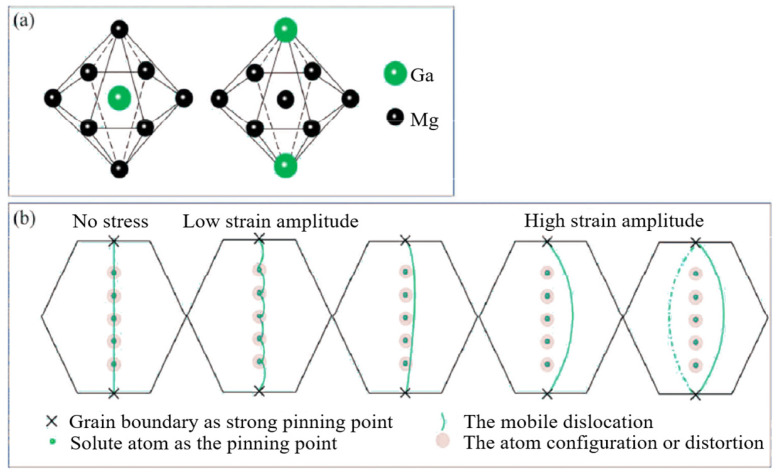
(**a**) Existent form of Ga atoms in α-Mg, and (**b**) improved G-L model [77].

Srikanth et al. [76] added Cu to AZ91 and found that the damping performance of a magnesium alloy increased with an increase in Cu content in a particular range. This phenomenon occurs because adding the Cu element increases the dislocation density and the range of the plastic deformation zone. The effect of the Y element on a ZK60 magnesium alloy was studied by Wang et al. [23]. They found that when the mass fraction of Y element as 2.5%, the magnesium alloy’s damping performance and mechanical properties could reach a reasonable level. This phenomenon is attributed to the formation of a new phase, Mg_3_Y_2_Zn_3_. On the one hand, Mg_3_Y_2_Zn_3_ purifies the magnesium matrix and reduces dislocation pinning when it is formed; on the other hand, the thermal expansion coefficients of Mg_3_Y_2_Zn_3_ and the magnesium matrix are pretty different, which leads to an increase in dislocation density. Huang et al. [77] analyzed the solid solution strengthening and damping properties of a Mg-Ga binary alloy. They found that adding Ga not only improved the mechanical properties of the magnesium alloy, but also optimized the damping properties of the magnesium alloy. Regarding the experimental results, the authors put forward an improved G-L dislocation model, as shown in Figure 7. Because of the great difference between the radii of the Ga atom and Mg atom, there are many lattice distortions and modulus mismatches the crystal. Dislocation will dissipate extra energy when interacting with them in motion, increasing damping performance.

During extrusion, the microstructure of Mg-4Li-3Al-0.3Mn alloys is refined and many nano-precipitates are produced, which reduces the pinning effect of solute atoms. In addition, according to the research of Lv et al. [78], magnesium alloys with a low Schmidt factor have a lower dislocation separation force. In contrast, a dispersed second phase appeared in the extruded-state alloy, so to sum up, the damping performance of Mg-4Li-3Al-0.3Mn alloy was also high in the extruded state. The addition of Mn and Al also promoted the formation of a precipitation phase and improved the damping and mechanical properties of the alloy. Wan et al. [79] also conducted similar research. They added Al to Mg97Zn1Y2 and found that the damping and mechanical properties of the magnesium alloy were improved. Adding the Al element can make more solute atoms dissolve into the matrix, thus reducing the pinning of dislocation motion and making dislocations easier to start. However, too much Al element will also lead to the formation of Mg_17_Al_12_, and this second phase serves as a strong pinning point to pin dislocations and reduce the damping performance of magnesium alloys.

### 3.3. Grain Orientation

Pure magnesium possesses many advantages, but its difficulty in forming at room temperature limits the development of magnesium alloys. There are only two independent slip systems in pure magnesium at room temperature, which is the fundamental reason for its poor plasticity [80]. Some scholars have found that the slip of non-basal surfaces of magnesium alloys can be opened at higher than 180 °C. This indicates the dominant role of deformation twinning in the deformation of magnesium alloys at room temperature. Deformation twinning is also usually considered an effective means to control the grain orientation of magnesium alloys. In recent years, many scholars have studied the effect of grain orientation on the damping performance of magnesium alloys. Grain orientation mainly affects the damping properties of magnesium alloys in two ways. On the one hand, favorable orientation can promote the basal plane slip of magnesium alloy; on the other hand, grain orientation that is conducive to dislocation initiation can reduce the critical shear stress and texture strength of the base surface. The stronger the basal intensity affected by the grain orientation, the worse its damping performance [81,82].

Zhou et al. [83] found that the stronger the basal texture intensity of a magnesium alloy, the smaller the chance of dislocation actuation under the same stress, and the lower the performance of the magnesium alloy. They took compressed samples in different compression directions and found that the damping properties of wrought magnesium alloys were anisotropic. Under high strain amplitude, the damping performance of the sample parallel to the extrusion direction is the best. Yan et al. [84] found that Mg-0.6Zr sheets rolled at high strain rates possess higher damping properties. The dynamically recrystallized grains rotate to weaken the preferred direction of the basal texture, resulting in a weaker basal texture [85]. So, the damping properties of the magnesium alloy are enhanced. 

Gan et al. [86] found that specimens oriented at 45° to the extrusion direction at high strain amplitudes had the best damping properties because the soft orientation of the base made it easier to activate dislocations. However, there was no significant difference in temperature-dependent damping performance for specimens with different orientations. Temperature is also a critical factor in the damping performance of alloys. K. Hazeli et al. [87] found a significant plateau region in temperature-dependent damping values after the addition of the Sr element to an AZ31 magnesium alloy. The addition of the Sr element decreases the solubility of Al in the Mg matrix, dislocations are allowed to move more easily and more dislocation systems are activated in the alloy. Twinning occurs at 180 °C, also leading to the formation of new textures. This study provides an idea for the design of Mg-Al-based high-performance Mg alloys containing Sr.

### 3.4. Grain Boundaries

According to the G-L dislocation theory, grain boundaries are strong pinning points for pinning dislocations. The smaller the grains, the larger the number of grain boundaries, the harder the dislocation is to activate, and the lower the damping performance of magnesium alloys. However, in some binary or even ternary magnesium alloys, the grains become very small due to the influence of alloying elements, and these alloys obtain better damping properties. It is difficult for dislocation theory to explain the major damping source of magnesium alloys, and no deformation twins have been found in these alloys [88]. Therefore, grain boundary relaxation has become a new damping source [89,90]. 

**Figure 8:**
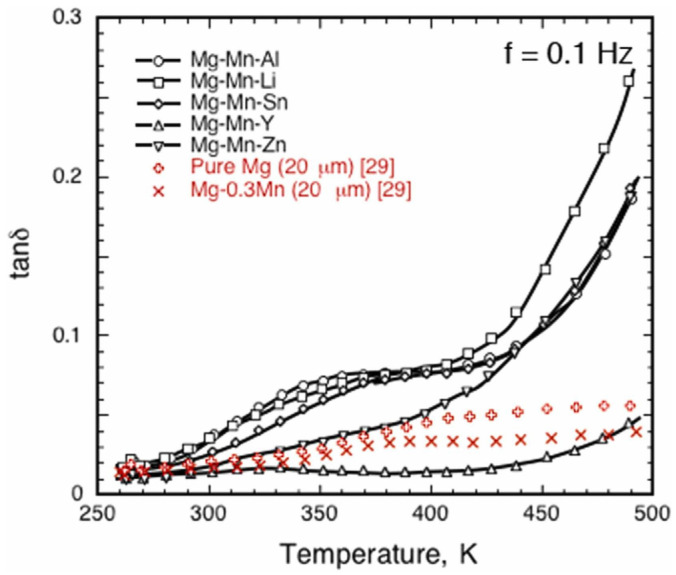
The variation in damping capacity, tanδ, as a function of temperature in extruded Mg-Mn ternary alloys [91].

Somekawa et al. [91] studied the grain boundary plasticity of a Mg-Mn ternary alloy at room temperature. The alloying elements always converge near the grain boundary, affecting both the grain boundary’s relaxation and damping properties. In previous studies, alloying elements were roughly divided into three categories, I (Al, Li, Mn, Sn), II (Ca, Y) and III (Ag, Zn) [92]. The first type of element will promote grain boundary relaxation, but the second type will hinder it. The third category is the same as the second but less prevented. This is consistent with the experimental results that are shown in Figure 8, and proves that grain boundary relaxation is one of the main damping mechanisms of extruded magnesium alloys.

## 4. Summary and Outlook

With the deepening of research of the damping property of magnesium alloys, the pursuit of higher damping properties and ensuring mechanical properties has become a new research goal. Currently, the design of magnesium alloys with high damping and high mechanical properties requires the synergy of multiple damping mechanisms. Now it is necessary to design a suitable dislocation movement structure, such as the LPSO phase. Dislocation in the LPSO phase is easier to start, which obviously improves the damping performance of magnesium alloys. At low temperatures, the damping mechanism of magnesium alloys is mainly based on the G-L dislocation theory. Energy dissipation can be achieved via dislocation movement between weak or strong pinning points. The interface is thermally activated at high temperatures, which also consumes extra energy at high temperatures. On these bases, some scholars have put forward an improved model of the G-L dislocation theory. The key to developing a damping magnesium alloy with an integrated structure and function is to understand the factors affecting the damping performance of magnesium alloys. Among the internal factors affecting the damping properties of magnesium alloys, the role of dislocation density, solute atoms, grain orientation and boundaries has been widely acknowledged. The density of dislocations should be moderate. If the density of dislocations is too small, it is difficult for dislocations to start and energy to dissipate. And if the density is too high, the dislocation line entanglement will be difficult to move. Adding rare earth elements (such as Y, Ga, Gd, etc.) to magnesium alloys has become an effective means to manufacture magnesium alloys with high damping and mechanical properties. The grain orientation can be adjusted to promote the sliding of the basal surface, which is also an effective way to improve the damping properties of magnesium alloys. Grain boundary relaxation is an important means to dissipate energy, but too many grain boundaries will enhance the pinning effect of dislocation. Excellent damping performance requires the cooperation of new or multiple damping mechanisms. This is a hot research topic that aims to discover and improve new damping mechanisms that can fully explain the damping properties of magnesium alloys. And it is also important to consider all the factors affecting damping properties and to design suitable damping magnesium alloys. 

## Data Availability

Not applicable.
